# 

**Published:** 1862-05

**Authors:** 


Chicago

New Series, Vol. 5, No. 5. Whole Series, Vol. 19, No. 5.

Dr R C Hamill
1305

### THE CHICAGO

### MEDICAL JOURNAL.

DANIEL BRAINARD, M. D.,
J. ADAMS ALLEN, M. D.,
Editors and Proprietors.

MAY, 1862.

CHICAGO:

WM. PIGOTT, BOOK AND JOB PRINTER, 130 CLARK STREET.

1862.

